# Ambulatory Care Visits to Pediatricians in Taiwan: A Nationwide Analysis

**DOI:** 10.3390/ijerph121114043

**Published:** 2015-11-02

**Authors:** Ling-Yu Yang, An-Min Lynn, Tzeng-Ji Chen

**Affiliations:** 1 Department of Pediatrics, School of Medicine, National Yang-Ming University, No. 155, Section 2, Linong Street, Taipei 112, Taiwan; 2 Department of Medical Education, Taipei Veterans General Hospital, No. 201, Section 2, Shi-Pai Rd., Taipei 112, Taiwan; 3 Division of Family Medicine, National Yang-Ming University Hospital, No. 152, Xinmin Rd., Yilan City, Yilan County 260, Taiwan; E-Mail: nashsaka@hotmail.com; 4 Department of Family Medicine, Taipei Veterans General Hospital, No. 201, Section 2, Shi-Pai Rd., Taipei 112, Taiwan; E-Mail: tjchen@vghtpe.gov.tw; 5 Department of Family Medicine, School of Medicine, National Yang-Ming University, No. 155, Section 2, Linong Street, Taipei 112, Taiwan

**Keywords:** Pediatricians, national health insurance, ambulatory visits

## Abstract

Pediatricians play a key role in the healthy development of children. Nevertheless, the practice patterns of pediatricians have seldom been investigated. The current study analyzed the nationwide profiles of ambulatory visits to pediatricians in Taiwan, using the National Health Insurance Research Database. From a dataset that was randomly sampled one out of every 500 records among a total of 309,880,000 visits in 2012 in the country, 9.8% (*n* = 60,717) of the visits were found paid to pediatricians. Children and adolescents accounted for only 69.3% of the visits to pediatricians. Male pediatricians provided 80.5% of the services and the main workforces were those aged 40–49 years. The most frequent diagnoses were respiratory tract diseases (64.7%) and anti-histamine agents were prescribed in 48.8% of the visits to pediatricians. Our detailed results could contribute to evidence-based discussions on health policymaking.

## 1. Introduction

Pediatricians play a requisite role in child and adolescent health care, including providing neonatal care, managing congenital or developmental abnormalities and pediatric diseases. Since it was established approximately 200 years ago, the specialty has continued to develop into a well-rounded and unique subset of knowledge-based content [1]. In Taiwan, the child health care indices are as good as they are in the USA in the aspects such as newborn mortality, infant mortality, under-5 mortality, and the mortality of the population aged 1 to 18 years [2]. However, Taiwan has also become and ranked among the lowest fertility countries in recent decades, synchronizing with the global trend in the decline of fertility rates [3]. The turning point came in 1984 when Taiwan first experienced that total fertility rates (TFR) went lower than the replacement level. Taiwan’s TFR decreased further from 1.80 to 0.89 between 1990 and 2010, which inevitably impacted on the pediatricians, not to mention that pediatricians also have to compete with other specialists such as family medicine specialists and otorhinolaryngologists [4]. In the United States, a National Ambulatory Medical Care Survey (NAMCS) has been conducted to survey who were providing care to America’s children [5] as well as the antimicrobial prescribing patterns for children [6,7,8,9]. The research on the antimicrobials prescribed by pediatricians have also been reported in some other countries [10,11,12]. In Taiwan, a previous study had investigated the role of pediatricians for children during 1999 to 2011 [13], and another one studied the common diagnosis in 2009 by collecting data from the National Health Insurance Research Database (NHIRD) [14]. Nevertheless, in many countries, including Taiwan, a nationwide survey on the demographic data of pediatricians and their practicing patterns is still lacking.

The current study aimed to study the characteristics of ambulatory visits to pediatricians on a nationwide base retrieving the record from Taiwan’s National Health Insurance (NHI) system in 2012. The variables analyzed included ages and genders of both patients and physicians, the procedures conducted, the diagnoses made, and the medications prescribed during these visits. The findings may present valuable information for future healthcare policymaking, and may deed as a comparison for longitudinal researches.

## 2. Methods

### 2.1. Database

The NHI program starting from 1995 in Taiwan has offered comprehensive health care to cover more than 99% of the country's residents. The National Health Insurance Administration of the Ministry of Health and Welfare has released all the de-identified claims data traced back to 1999 for academic research in the form of the National Health Insurance Research Database (NHIRD) [15]. The conduct of the study had been approved by the institutional review board (IRB) of Taipei Veterans General Hospital, Taipei, Taiwan (2013-04-005E). Because of anonymized data that are publicly available on application, our study is exempt from full IRB review.

### 2.2. Study Population

We performed the descriptive and cross-sectional study by accessing the files sampled from those of the year 2012 (S_CD20120.DAT and S_OO20120.DAT of NHIRD). The dataset “CD” means collection of all the outpatient visit files, on the other hand, the dataset "OO" refers to the outpatient order files. These two collections of files, containing a total of 619,760 medical records, were acquired by a 0.2% sampling ratio from the CD and OO datasets for 2012, excluding dentistry and traditional Chinese medicine. Each record comprised the patient’s identification number, sex, birth date, date of consultation, medical facility, the specialty of the physician consulted, and up to three diagnosis codes as defined by the International Classification of Diseases, Ninth Revision, Clinical Modification (ICD-9-CM).

The details of the medical records of 60,717 ambulatory visits to pediatricians were unpacked for analysis. The National Health Insurance Administration also listed reimbursable drugs with additional coding in the Anatomical Therapeutic Chemical (ATC) classification system [16]. The basic data of the contracted medical care institutions offered the status of accreditation: academic medical center, metropolitan hospital, local community hospital, or physician clinic. The diagnoses, procedures and prescriptions of medications made during the visits were analyzed and compared among different levels of hospitals or clinics.

### 2.3. Statistical Analysis

The programming software Perl version 5.20.2 was used for data processing, and then regular descriptive statistics were displayed.

## 3. Results

Of the 619,760 ambulatory visits collected in 2012, 9.8% (*n* = 60,717) were attended by pediatricians—ranking the fourth among all the physician specialties (Table 1). Pediatricians took charge of 5.1% of insurance cost claimed, amounting to an estimate of NT$309 billion.

**Table 1:** Ambulatory visits covered by Taiwan’s National Health Insurance in 2012, stratified by specialty (sampling rate: 1/500).

Specialty	Number of Visits (%)	Cost Claimed (%)	Average Cost Claimed per Visit
Family medicine	116,551 (18.8)	51,157,561 (8.3)	439
Internal medicine	70,615 (11.4)	42,547,879 (6.9)	602
Otorhinolaryngology	67,881 (11.0)	29,718,395 (4.8)	438
Pediatrics	60,717 (9.8)	31,574,986 (5.1)	520
Ophthalmology	37,692 (6.1)	25,422,291 (4.1)	674
Obstetrics& Gynecology	35,697 (5.8)	19,674,700 (3.2)	551
Others	230,607 (37.2)	418,023,780 (67.6)	1813
Total	619,760 (100.0) *	618,119,592 (100.0)	997

* The percentage of the ambulatory visits and cost claimed by specialty had been rounded, therefore the % in each of the table didn’t give a total of 100% just.

**Figure 1:**
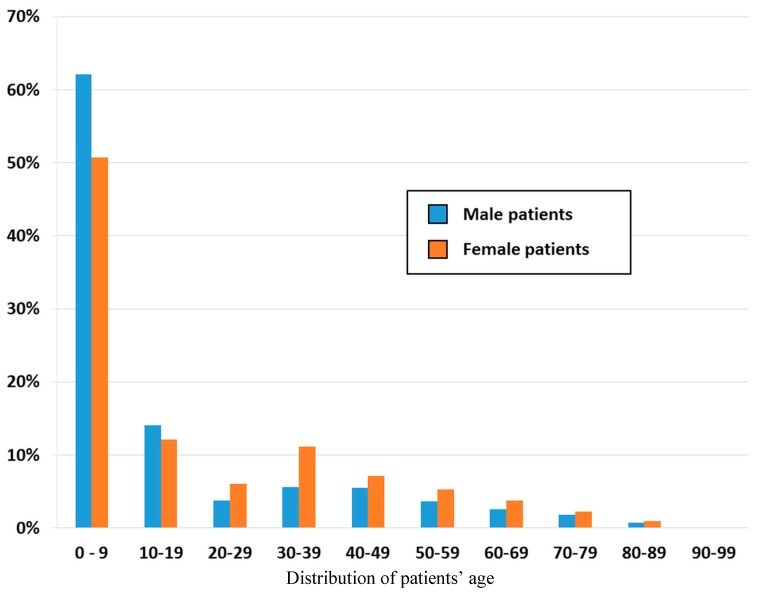
Age and sex distribution of the patients who visited pediatricians, data from Taiwan’s National Health Insurance in 2012 (sampling rate: 1/500).

Among the patients paying the ambulatory visits to pediatricians, 52% were male, and 48% were female. Stratifying the data by age group we found that the group of patients aged 0–10 years accounted for the highest proportion (56.2%, *n* = 34,111) of ambulatory visits to pediatricians in both genders (male: 62.1%, *n* = 18,089; female: 50.7%, *n* = 16,022), followed by the patients aged 10–19 years (13.1%, *n* = 7963) in both genders (male: 14.1%, *n* = 4118; female: 12.1%, *n* = 3845). Children and adolescents accounted for nearly seventy percent (69.3%) of the ambulatory visits to pediatricians, while the other older groups, ≥20 years of age, accounted for 30.7% (*n* = 18,643) of the visits. There were more visits paid by female patients than by male patients (female, *n* = 11,712; male, *n* = 6931) (Figure 1).

**Figure 2:**
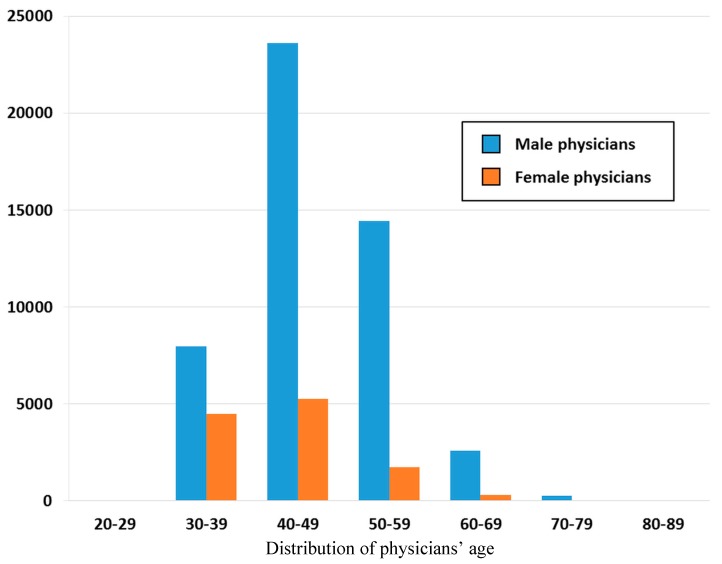
Age and sex distribution of pediatricians, data from Taiwan’s National Health Insurance in 2012 (sampling rate: 1/500).

The number of ambulatory visits in reference to the pediatricians’ gender and age is presented in Figure 2. The patients, at whatever age groups, showed to pay more visits to male pediatricians than to female pediatricians. The pediatricians aged 40–49 years, in both genders, attended to the highest proportion of ambulatory visits (male = 23,603, female = 5255), as compared with the other age groups of pediatricians. There were fewer pediatricians in the male group aged 30–39 years (*n* = 7954), as compared with the same gender group of pediatricians aged 40–49 (*n* = 25,603) and 50–59 years (*n* = 14,433).

**Figure 3:**
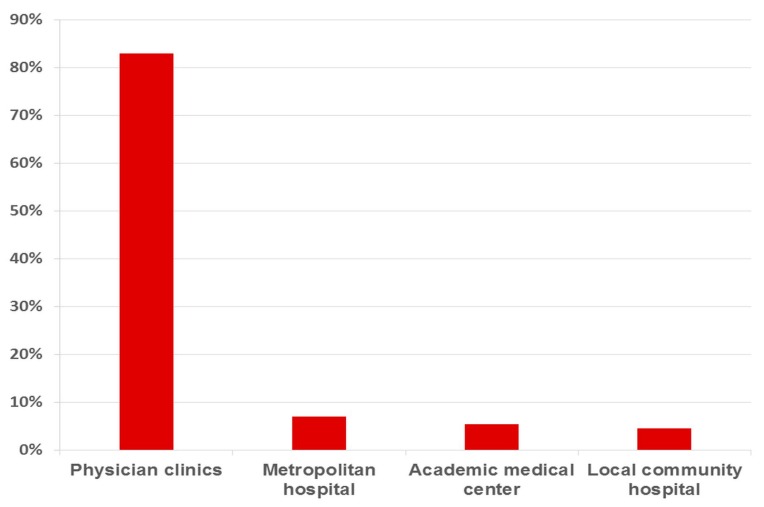
Pediatricians’ affiliation, stratified according to healthcare facility accreditation, data from Taiwan’s National Health Insurance in 2012 (sampling rate: 1/500).

This study showed that practitioners remained the most important ambulatory care providers and managed 83.0% (*n* = 50,448) of the ambulatory care visits made by pediatricians (*n* = 60,717) from 619,760 sampled ambulatory visits in Taiwan, followed by metropolitan hospitals (7.0%, *n* = 4194), academic medical centers (5.4%), and local community hospitals (4.6%) (Figure 3).

We further analyzed the age distribution of the physicians, stratified in accordance with healthcare facility accreditation (Table 2). The ambulatory visits in the clinics were managed mostly by the physicians aged 40–49 years (*n* = 24,559), followed in frequency by the 50–59 years group (*n* = 13,758). In the academic medical centers, however, physicians aged 40–49 (*n* = 1321) and 50–59 (*n* = 1104) years showed to have managed similar numbers of ambulatory visits.

**Table 2:** Pediatricians’ age affiliation, stratified according to healthcare facility accreditation, data from Taiwan’s National Health Insurance in 2012 (sampling rate: 1/500).

Facility Level	20–29 Years	30–39 Years	40–49 Years	50–59 Years	60–69 Years	70–79 Years	80–89 Years	Total
Physician clinics	20	9538	24,559	13,758	2307	251	12	50,448 *
Local community hospital	3	827	1223	578	167	25	2	2825
Metropolitan hospital	7	1525	1755	734	171	0	2	4194
Academic medical center	21	545	1321	1104	240	19	0	3250
Total	51	12,435	28,858	16,174	2885	295	16	60,717

* There were three “unknown” in the physician clinics.

Among the ambulatory visits to pediatricians, 65.9% (*n* = 40,014) produced only one diagnosis. According to the major disease category, respiratory diseases were the most frequently coded diagnostic group (*n* = 39,305, 64.7%), followed by non-specific symptoms and signs, and other ill-defined conditions (*n* = 4872, 8.0%). The first diagnosis code in every medical record was analyzed and assembled into a list of the top 10 most common diagnosis groups (Table 3).

Acute upper respiratory infection was the most common diagnosis (22.2%), followed in frequency by acute bronchitis & bronchiolitis (11.6%), acute sinusitis (5.1%), acute tonsilitis (5.0%) and acute nasopharyngitis (4.3%). The ranking of the diagnosis groups differed with accredited hospital level. For example, in academic medical centers, asthma was the most common diagnosis (*n* = 205, 0.3%), followed in order by allergic rhinitis (*n* = 182, 0.3%), epilepsy (*n* = 131, 0.2%), acute bronchitis & bronchiolitis (*n* = 121, 0.2%), infant and child health supervision (*n* = 121, 0.2%), acute upper respiratory infections (*n* = 104, 0.2%), *bulbus cordis* anomalies & anomalies of cardiac septal closure (*n* = 90, 0.2%), bronchopneumonia (*n* = 76, 0.1%), symptoms related to nutrition, metabolism & development (*n* = 74, 0.1%), and other endocrine disorders (*n* = 73, 0.1%).Overall, the most common procedures performed during ambulatory visits to pediatricians were chest view (0.7%, *n* = 425), complete blood count (0.7%, *n* = 404), humidity or aerosol therapy-time (0.6%, *n* = 383), white blood cell differential count (0.6%, *n* = 374), and general urine examination (0.5%, *n* = 304) (Table 4).

The utilization of the procedures also varied with different ambulatory care settings. For example, complete blood count was the most common procedure applied in medical centers, while humidity or aerosol therapy-time was performed most frequently in physician clinics. Of the ambulatory visits to pediatricians, 92.5% (*n* = 56,169) were managed by medication. Only 10.6% of the visits were given with a single drug prescription, whereas approximately 81.9% of the visits were prescribed with two or more drugs. Moreover, 50.5% had the prescriptions of four or more drugs. The most commonly prescribed medications were anti-histamine (48.8%), expectorants (32.0%) and cough suppressants (26.5%; Table 5).

**Table 3:** Ambulatory visits to pediatricians, data from Taiwan’s National Health Insurance in 2012, stratified by disease group and hospital level (sampling rate: 1/500, the percentage of diagnoses had been rounded).

ICD9CM *	Diagnosis Group	Total N = 60,717	Academic Medical Center N = 3250	Metropolitan Hospital N = 4194	Local Community Hospital N = 2825	Physicians Clinics N = 50,448
465	Acute upper respiratory infections	13,461 (22.2)	104 (0.2)	287 (0.5)	271 (0.5)	12,799 (21.0)
466	Acute bronchitis & bronchiolitis	7041 (11.6)	121 (0.2)	469 (0.8)	348 (0.6)	6103 (10.1)
461	Acute sinusitis	3072 (5.1)	58 (0.1)	120 (0.2)	86 (0.1)	2808 (4.6)
463	Acute tonsilitis	3030 (5.0)	37 (0.1)	163 (0.3)	112 (0.2)	2718 (4.5)
460	Acute nasopharyngitis	2597 (4.3)	13 (0.0) **	34 (0.1)	33 (0.1)	2517 (4.1)
462	Acute pharyngitis	2580 (4.2)	52 (0.1)	118 (0.2)	146 (0.2)	2264 (3.7)
558	Other noninfectious gastroenteritis and colitis	2032 (3.3)	58 (0.1)	120 (0.2)	61 (0.1)	1793 (2.9)
464	Acute laryngitis & tracheitis	1688 (2.8)	9 (0.0) **	20 (0.0) **	17 (0.0) **	1642 (2.7)
V20	Health supervision of infant or child	1644 (2.7)	121 (0.2)	239 (0.4)	301 (0.5)	983 (1.6)
780	Non-specific symptoms	1544 (2.5)	68 (0.1)	170 (0.3)	94 (0.2)	1212 (2.0)

* The International Classification of Diseases, 9th Revision, Clinical Modification; ** The value that had been rounded still less than 0.001 (0.1%).

**Table 4:** Top ten procedures and laboratory tests prescribed by pediatricians, data from Taiwan’s National Health Insurance in 2012 (sampling rate: 1/500 sampling).

NHI Code *	Procedure	No. of Visits	%
32001C	Chest view (including each view of chest film)	425	0.7%
08001C	Complete blood count	404	0.7%
57021C	Humidity or aerosol therapy- time	383	0.6%
08013C	White blood cell differential count	374	0.6%
06012C	General urine examination	304	0.5%
09026C	S-GPT/ALT	225	0.4%
09005C	Blood glucose	212	0.3%
57110C	Blood sampling	208	0.3%
18007B	Doppler color flow mapping	178	0.3%
09015C	Serum creatinine	176	0.3%

* Taiwan’s National Health Insurance code.

**Table 5:** Top ten drug classes prescribed by pediatricians, data from Taiwan’s National Health Insurance in 2012 (sampling rate: 1/500).

ATC Code *	Drug Classification	No. of Visits	%
R06A	Antihistamines for systemic use	29,608	48.8
R05C	Expectorants	19,457	32.0
R05D	Cough suppressants	16,091	26.5
M01A	Anti-inflammatory, non-steroids	15,003	24.7
R03C	Adrenergics for systemic use	14,865	24.5
N02B	Other analgesics and antipyretics	14,021	23.1
R01B	Nasal decongestants for systemic use	11,451	18.9
R05F	Cough suppressants & expectorants combinations	9142	15.1
A03A	Drugs for functional bowel disorders	7640	12.6
R03D	Other systemic drugs for obstructive airway diseases	6463	10.6

* Anatomical Therapeutic Chemical code.

## 4. Discussion

The present study confirmed the contribution of physician clinics as the main providers of the ambulatory care to attend to 83.0% (*n* = 50,448) of the visits to pediatricians. The primary medical practitioners took the heaviest responsibilities in managing the need for pediatric care. Namely, the pattern of ambulatory visits met the expectation of primary medical care for minor conditions.

The pediatricians are well-disciplined as a specialty to offer medical care for people before their adulthood, including neonates, infants, toddlers, pre-schools, school-ages, and adolescents. Therefore, in usual case, the patients who visit the pediatricians should fall into the range aged 0–19 years. However, we noticed a number of people who visited the pediatricians even after their twenties, which occurred more in female patients than in male patients. The reasons may be multiple. First, the female patients tend to care more about their own health condition and have more health seeking behaviors [17,18,19]. Second, mothers are more likely to bring their children to see doctors, therefore have more opportunities to visit the pediatricians than fathers do. Further, as listed in Table 3, upper respiratory infection is the most common diagnosis and might run the risk of group infection. That means the family who bring the children may also need medical care meanwhile.

In our study, more visits were paid to male physicians than to female physicians. The gender disparity turned more apparent with age of the physicians (Figure 2) possibly because fewer women were educated to become physicians in the past [20] (p.18, pp.161–162). Another finding was that the majority of visits went to pediatricians aged 40–49 years, coupled with the fact that most of the visits were managed in the clinics. The pediatricians aged 40–49 years were out of question regarded as the principal supply of workforce in the clinics. Among the number of investigations on manpower of pediatricians, a report from Taiwan has indicated an obvious shift of more and more workforce of the pediatricians from hospitals to local clinics, in view of the incessant drop of fertility condition that rendered insufficiency or aging of manpower in hospitals [21]. Similar phenomenon was also observed in obstetrician-gynecologists [22].

In Taiwan, unbalanced development among different medical specialties has always been a problem. According to a previous report, new applicants for pediatric residency training and candidates for licensure examination of pediatricians have both decreased in the recent years [21]. The rural-urban inequality of practicing pediatricians has also deteriorated due to the decrease of pediatricians [23]. The insufficient medical care was estimated to affect approximately 27% of the pediatric population [24]. Moreover, among the total of 319 townships in this island, 132 had no pediatrician at all. A longitudinal, retrospective study [25] was conducted and the result inferred that the amount of pediatricians would continue to decrease because of the reduced child populations, thus to increase the workload of each pediatrician.

As demonstrated in Table 3, respiratory diseases were the most common cause for ambulatory visits to pediatricians. This finding was consistent with a previous report [14], and could thus help explain why some common procedures such as blood examination or chest X-ray (Table 4) were relatively underused. URI does not require laboratory evidence for diagnosis and the children are usually intolerant of invasive procedures. Chest X-rays are not necessary for URI, either, unless there is concern about lower respiratory tract involvement.

Regarding the prescription of medication, anti-histamine agents were prescribed the most often, accounting for nearly half of the visits (48.8%). Symptomatic agents including non-steroid anti-inflammatory drugs, antipyretics, expectorants and cough suppressants were all commonly used for URI. Prescribing antibiotics in the pediatric population has become a global issue, especially concerning their overuse for URI. In the United States, the prescriptions of antibiotics for children with URI has decreased in these years [26,27,28]. Intervention by strict criteria, enforcement of national guidelines and community-wide campaign could have influences in antibiotics prescribing for children [29,30,31]. However, the low overall consumption of antibiotics is by no means an indicator of appropriate pediatric antibiotic prescribing [12]. American Academy of Pediatrics has published an article and presented that broad-spectrum antibiotic prescribing accounts for 50% of antibiotic use in ambulatory pediatrics in respiratory conditions for which antibiotics are not indicated [32]. In Taiwan, we have guideline on community acquired pneumonia and influenza infection for children [33]. Ever since the NHI program restricted the use of antibiotics for URI in 2001, antibiotics never appear among the top 10 most prescribed medications. However, the appropriate use of antibiotics still needs continuing investigation.

The resources used in this study, which were compiled by the National Health Insurance Administration, impose certain limitations on our analysis. For instance, the results do not include self-paid procedures or medicines, such as expensive vaccines and medicine. In Taiwan, atypical pneumonia is not uncommon [34], but the use of macrolides (e.g., azithromycin) is self-paid except for erythromycin, unless laboratory confirmation is carried out. As for non-publicly funded vaccines, conjugated pneumococcal vaccine must be paid with coverage by some local governments in Taiwan. Nevertheless, since the NHI program pays for most diseases and preventive care, the above issues no longer have a significant impact on the pattern of ambulatory visits recorded in this study, although it should also be noted that our figures do not present a complete picture. Second, the pediatricians tend to make tentative rather than final diagnoses for claims at ambulatory setting. Therefore, the accuracy of the diagnoses is not guaranteed. Third, our analyses using visit-based sampling datasets might fail to reveal the comorbidities and subsequent status of the patients studied.

## 5. Conclusions

In Taiwan, male pediatricians aged 40–49 years provided most of the ambulatory care services. Physician clinics handled four-fifths of the ambulatory visits. Respiratory tract diseases accounted for 64.7% of the visits to pediatricians, and the majority of them were managed at the local clinics. In addition, prescribing multiple drugs for symptomatic patients with URI should be further investigated to evaluate their potential benefits or damage, and at the same time to appropriately allocate the costs.
